# Molecular Characterization of a Novel 1,3-α-3,6-Anhydro-L-Galactosidase, Ahg943, with Cold- and High-Salt-Tolerance from *Gayadomonas joobiniege* G7

**DOI:** 10.4014/jmb.2008.08017

**Published:** 2020-08-28

**Authors:** Ju Won Seo, Maral Tsevelkhorloo, Chang-Ro Lee, Sang Hoon Kim, Dae-Kyung Kang, Sajida Asghar, Soon-Kwang Hong

**Affiliations:** 1 Department of Bioscience and Bioinformatics, Myongji University, Yongin 7058, Republic of Korea; 2 Department of Animal Resources Science, Dankook University, Cheonan 31116, Republic of Korea

**Keywords:** 1,3-α-3,6-anhydro-L-galactosidase, α-neoagarooligosaccharide hydrolase, Gayadomonas joobiniege G7, cold- and salt-tolerant, monomer

## Abstract

1,3-α-3,6-anhydro-L-galactosidase (α-neoagarooligosaccharide hydrolase) catalyzes the last step of agar degradation by hydrolyzing neoagarobiose into monomers, D-galactose, and 3,6-anhydro-Lgalactose, which is important for the bioindustrial application of algal biomass. Ahg943, from the agarolytic marine bacterium *Gayadomonas joobiniege* G7, is composed of 423 amino acids (47.96 kDa), including a 22-amino acid signal peptide. It was found to have 67% identity with the α-neoagarooligosaccharide hydrolase *Zg*AhgA, from *Zobellia galactanivorans*, but low identity (&lt; 40%) with the other α-neoagarooligosaccharide hydrolases reported. The recombinant Ahg943 (rAhg943, 47.89 kDa), purified from *Escherichia coli*, was estimated to be a monomer upon gel filtration chromatography, making it quite distinct from other α-neoagarooligosaccharide hydrolases. The rAhg943 hydrolyzed neoagarobiose, neoagarotetraose, and neoagarohexaose into D-galactose, neoagarotriose, and neoagaropentaose, respectively, with a common product, 3,6- anhydro-L-galactose, indicating that it is an exo-acting α-neoagarooligosaccharide hydrolase that releases 3,6-anhydro-L-galactose by hydrolyzing α-1,3 glycosidic bonds from the nonreducing ends of neoagarooligosaccharides. The optimum pH and temperature of Ahg943 activity were 6.0 and 20°C, respectively. In particular, rAhg943 could maintain enzyme activity at 10°C (71% of the maximum). Complete inhibition of rAhg943 activity by 0.5 mM EDTA was restored and even, remarkably, enhanced by Ca^^2+^^ ions. rAhg943 activity was at maximum at 0.5 M NaCl and maintained above 73% of the maximum at 3M NaCl. *K_m_* and *V_max_* of rAhg943 toward neoagarobiose were 9.7 mg/ml and 250 μM/min (3 U/mg), respectively. Therefore, Ahg943 is a unique α-neoagarooligosaccharide hydrolase that has cold- and high-salt-adapted features, and possibly exists as a monomer.

## Introduction

Agarose is a major constituent of agar, the main component of red algal biomass. Agar is a heterogeneous hydrophilic colloidal polysaccharide composed of alternately linked 3-*O*-linked β-D-galactopyranose (G) and 4-*O*-linked α-L-galactopyranose (L) [1, 2]. Agarose is a main component of agar, where L is substituted with 3,6‐anhydro‐L‐galactose (L-AHG), forming a linear polymer with an average molecular mass of 120,000 *Da* [3]. Agar has been used as a dietary food and gelling agent for a long time in many Asian countries and has been generally recognized as safe by the United States Food and Drug Administration. Owing to the unique properties of thermal hysteresis in the sol-to-gel transition and its structural stability, agar has been widely used in bacterial plate culture as well as in electrophoretic and chromatographic supporting materials [4].

Recently, marine algal biomass has been highlighted in many studies because it is a valuable and sustainable resource that can substitute fossil-based chemical feed stocks, including petroleum [5]. In addition, agar can be used as a supporting material for enzyme or bacterial immobilization to enhance the stability of the system, which allows long-term operation [6, 7]. Agar can also be used to manufacture biodegradable polymers, such as bioplastics [8], and can be used in wet-fiber [9], eco-friendly biocleaning processes [10] as well as in medical treatments, such as microencapsulation [11], drug delivery [12], and bone generation [13]. In this sense, agar is expected to be widely used in the food and chemical industry as well as in the medical field.

**Fig. 1:**
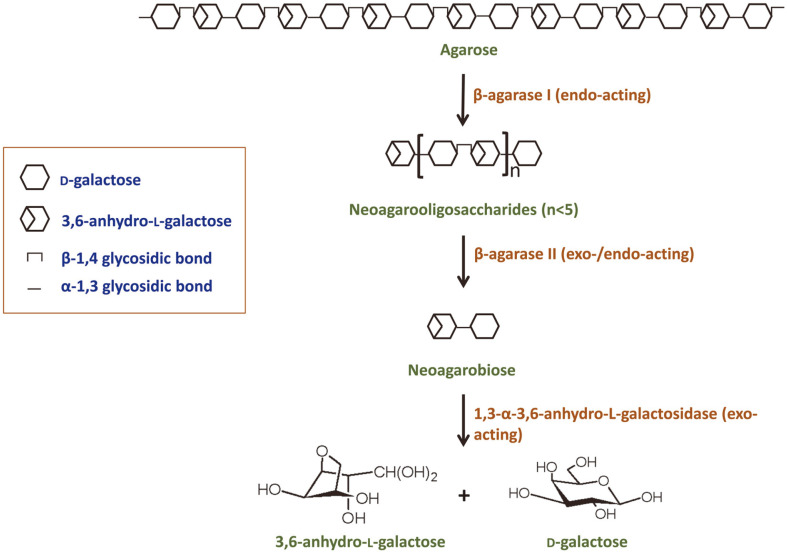
A simplified scheme for agar degradation by β-agarases in nature. In nature, agar is mainly broken down in via of β-agarolytic pathway. Polymeric agar is successively decomposed to disaccharides, neoagarobiose, by various β-agarases, which are further degraded to monosaccharides, D-galactose and 3,6-anhydro-L-galacotse, by 1,3-α-3,6-anhydro-Lgalactosidase including α-neoagarobiose/α-neoagarooligosaccharide hydrolases.

Agar can be degraded by chemical treatments or enzymatic hydrolysis for industrial applications. Because the former requires eco-unfriendly harsh reaction conditions and results in a lot of waste and unwanted toxic sugar derivatives, such 5-hydroxymethyl furfural, the latter process is generally preferred [14]. Agar is hydrolyzed via two routes; the α-agarolytic pathway mediated by α-agarases acting on the α-1,3 linkage, and the β-agarolytic pathway mediated by β-agarases acting on the β-1,4 linkage [4, 15]. Compared to many agarases involved in the β-agarolytic pathway being reported, only five α-agarases involved in the α-agarolytic pathway have been identified, which implies that the β-agarolytic pathway is a major route for agar degradation in nature [16]. In the β-agarolytic pathway (Fig. 1), β-agarase I degrades agar into large neoagarooligosaccharides (NAOSs), which are further hydrolyzed by β-agarase II into neoagarobiose. These NAOs, including neoagarobiose, have G at their reducing ends. Finally, neoagarobiose is completely degraded into monomers G and L-AHG by 1,3-α-3,6-anhydro-L-galactosidase, including α-neoagarobiose hydrolase (α-NABH) and α-NAOS hydrolase (α-NAOSH), for catabolic use by microorganisms. Therefore, several kinds of hydrolytic enzymes work together to metabolize agar in nature, which is the same principle as in the industrial application of agar. NAOSs and monomers (G and L-AHG) have various biological functions and are used for important chemical feed stocks [15, 16]. To date, many β-agarase I/II have been reported, but only a few α-NABH/α-NAOSH have been identified. Therefore, there is a need to accumulate information regarding α-NABH/α-NAOSH.

*Gayadomonas joobiniege* G7 is an agar-degrading marine bacterium belonging to a novel genus identified from coastal seawater in Korea [17]. Genomic sequencing revealed that it has many genes encoding hydrolytic enzymes for the complete breakdown of sulfated algal polysaccharides, and two novel α-NAOSHs, Ahg558 [18] and Ahg786 [19], were identified. In this study, we describe the biochemical identification of Ahg943 from *G. joobiniege* G7, which is unique among the reported α-NAOSHs.

## Materials and Methods

### Bacterial Strains and Culture Conditions

*G. joobiniege* G7 (ATCC BAA-2321 = DSM25250^T^ = KCTC23721^T^) was used as a genomic source for *ahg943* (NCBI reference sequence: WP_017446943) [20]. *Escherichia coli* ER2566 (New England Biolabs, USA) and plasmid pET28(a)+ were used for gene cloning and expression. *G. joobiniege* G7 and *E. coli* ER2566 were cultured in artificial sea water medium [17] and Luria-Bertani (LB) medium at 37°C, respectively. Kanamycin (final 50 μg/ml) was added to maintain the plasmid stability of the transformant.

### Chemicals

LB medium, isopropyl-β-D-thiogalactoside (IPTG), and kanamycin were purchased from Duchefa Biochemie (The Netherlands), and agarose and *pfu* polymerase were obtained from Enzynomics Co., Ltd. (Korea). The infusion HD cloning kit, Talon resin, and DNA modifying enzymes were purchased from Takara Bio (Japan), and the silica gel plates (60G F_254_) for thin layer chromatography were obtained from Merck KGaA (Germany). Primers for polymerase chain reaction (PCR) were synthesized by Genotech (Korea). NAOSs were purchased from DyneBio Inc. (Korea). Other chemicals were purchased from Sigma-Aldrich Corporation (USA).

### Cloning of the *ahg943* Gene

The in-fusion cloning method by PCR was used to obtain the *ahg943* gene sequence from the genomic DNA of *G. joobiniege* G7. The 1,206 bp DNA fragment encoding the entire mature form of Ahg558 without a signal sequence was amplified by PCR using the following primers: forward primer (5′-CGCGCGGCAGCCATATGG GGCAAACAATTGGAGC-3′; NdeI site is underlined) and reverse primer (5′-GCTCGAATTCGGATCCTTT TTACTTATAATTTTGGT-3'; BamHI site is underlined). The PCR reaction mixture (50 μl) containing template DNA (1 μl), 10 pmole of forward and reverse primer (1.5 μl each), 10 mM dNTPs (4 μl), 10x *pfu* buffer (5 μl), *pfu* polymerase (1 μl), and distilled water (DW, 36 μl) was reacted for 40 cycles, as previously described [18] in a PCR Thermal Cycler Dice Gradient (Takara Bio). The PCR products were purified using a PCR purification kit (Qiagen, Netherlands), digested with NdeI and BamHI restriction enzymes, then cloned into the pET28(a)+ vector using an in-fusion HD cloning kit (Takara Bio), yielding the recombinant plasmid pET28(a)-Ahg943. The nucleotide sequence of the *ahg943* finally cloned was confirmed by nucleotide sequencing, which showed no error.

### Expression and Purification of Recombinant Ahg943 Protein (rAhg943)

*E. coli* ER2566/pET28(a)-Ahg943 was inoculated in LB medium (3 ml) containing kanamycin (final 50 μg/ml) and incubated for 16 h at 37°C with shaking at 180 rpm. The pre-culture was transferred to a new LB medium (100 ml) containing kanamycin, which was further cultured under the same conditions. IPTG (final concentration of 0.5 mM) was added to the culture broth when the optical density at 600 nm (OD_600_) reached 0.5, and the overexpression of rAhg943 was induced for 16 h at 16°C. The cells were harvested by centrifugation at 10,000 ×*g* for 10 min, resuspended in 5 ml of binding buffer (50 mM Tris-HCl, 300 mM NaCl, pH 8.0), then disrupted by ultrasonic crushing (output control 4, duty cycle 40%) using a Sonifier 450 (Branson Ultrasonics Corp., USA) in an ice-water bath. The cell-free extract was prepared by centrifugation of the sonicated sample at 15,000 ×*g* for 30 min at 4°C and applied to TALON affinity column chromatography to purify the rAhg943 protein containing 6xHis-tag at its N-terminal. After washing the column twice with a binding buffer containing 5 mM imidazole, the rAhg943 protein was eluted with a binding buffer containing 200 mM imidazole. The eluate containing rAhg943 was dialyzed with 1 L of dialysis buffer (50 mM Tris, 100 mM NaCl, pH 8.0) for 12 h at 4°C, and then used for further experiments. The purity and molecular mass of the purified protein were analyzed by 0.1% sodium dodecyl sulfate-12% polyacrylamide gel electrophoresis (SDS-PAGE), as previously described [21]. The protein concentration was calculated using the Bradford method [22].

### Determination of Molecular Weight by Gel Filtration Chromatography

The molecular weight of the purified rAhg943 was determined by gel filtration chromatography using the ÄKTA-FPLC system (GE Healthcare Life Sciences, USA). A Superose 12 10/300 GL column was used and 50 mM Tris-HCl (pH 8.0) containing 100 mM NaCl was applied as the mobile phase with a flow rate of 0.5 ml/min at 15°C. The eluted protein was monitored using a UV detector at 280 nm.

### Measurement of Enzyme Activity Using the 3,5-Dinitrosalicylic Acid (DNS) Method

The enzyme activity of rAhg943 was estimated by the DNS method [23] by measuring the amount of reducing sugars released from the substrates. The reaction mixture (500 μl) contained 25 μg of protein (10 μl) and 1,000 μg of neoagarobiose (10 μl) in 0.5 M citrate buffer (pH 6.0). After an enzyme reaction at 20°C for 5 h, 500 μl of DNS solution (6.5 g of DNS, 325 ml of 2 N NaOH, and 45 ml of glycerol in 1 L DW) was added, and the mixture was heated in a boiling water bath for 10 min, then cooled in an ice-water bath for 10 min. The enzyme activity was measured at 540 nm (A_540_) using a Spectronic Unicam Genesys 8 Spectrophotometer (Thermo Scientific, USA). The blank sample contained 10 μl of DW instead of the enzyme. D-galactose was used as a reference reducing sugar for standard curve calibration. Enzyme activity (unit) was defined as the amount of enzyme that produced 1μmol of D-galactose per minute.

### Biochemical Characterization of rAhg943

The DNS method was used to investigate the optimal conditions for enzyme activity, such as temperature, pH, and metal ion concentrations. For the pH profile, the enzyme reaction was performed in 50 mM citrate buffer (pH 3.0-6.0), MOPS buffer (pH 6.0-7.0), Tris-HCl buffer (pH 7.0-9.0), and glycine buffer (pH 9.0-10.0) at 20oC for 5 h. For the temperature profile, the enzyme reaction was carried out in 50 mM citrate buffer (pH 6.0) containing 5 mM NaCl from 10°C to 60°C in 10°C intervals for 5 h. Finally, the effects of metal ions (CaCl_2_, CoCl_2_, CuCl_2_, FeCl_2_, KCl, MgCl_2_, MnCl_2_, NaCl, NiCl_2_, and ZnCl_2_) and EDTA on enzyme activity were tested in 50 mM citrate buffer (pH 6.0) in the presence of each chemical (final concentration of 5 mM) at 20°C for 5 h. The effect of NaCl on enzyme activity was measured under the same conditions at 0-3 M NaCl.

### Determination of the Kinetic Parameters

The kinetic parameters of rAhg943 were determined toward neoagarobiose by the DNS method. The hydrolysis reaction (total volume, 500 μl) by rAhg943 enzymes (25 μg) was performed in 50 mM citrate buffer (pH 6.0) containing 5 mM CaCl_2_ at 20°C for 5 min. The concentration of the substrate was set in the range of 0.2-1.2 mg/ml. Kinetic parameters (*K*_m_ and *V*_max_) were calculated using the Lineweaver-Burk plot [24].

### Thin-Layer Chromatography (TLC) of Hydrolyzed Products 

The hydrolyzates of NAOSs by rAhg943 were analyzed by TLC. The reaction mixture (50 μl) contained 500 μg of NAOS (neoagarobiose, neoagarotetraose, or neoagarohexaose) and 25 μg of enzyme in 50 mM citrate buffer (pH 6.0) containing 5 mM CaCl_2_. After enzyme reaction at 20°C for 24 h, the hydrolyzates were loaded onto TLC silica gel plates (60G F_254_) and developed with a solvent (n-butanol:ethanol:water = 3:1:1, v/v). The separated spots were visualized by spraying with 20% (v/v) sulfuric acid in ethanol then heating at 120°C for 15 min.

### Mass Analysis of Hydrolyzed Products 

The hydrolyzates of NAOSs by rAhg943 were prepared in the same manner as in the TLC analysis. The reactant was dried in an Eyela centrifugal evaporator CVE-2000 (EYELA, Japan). The methanol extract of the dried sample was analyzed using LC-TOF-MS (JMS-T100LPEOL Ltd, Japan). Electrospray ionization (ESI) conditions for the positive mode were as follows: orifice 1, 80 V; orifice 2, 5 V; ring lens, 15 V; ion guide RF, 2000 V; detector, 2,500 V. Orifice 1 was operated at a temperature of 80°C and the temperature of the desolvating chamber was 250°C. Mass spectra in the *m/z* range of 70-1020 were obtained using ESI in the positive-ion mode.

### Sequence Alignment, Phylogenetic Tree Construction, and Secondary Structure Analysis

The Clustal Omega program (https://www.ebi.ac.uk/Tools/msa/clustalo/) was used for sequence alignment of the Ahg943 amino acid sequence. The neighbor-joining (NJ) method [25] in the Mega 6 program [26] was adopted for constructing the phylogenetic tree. The tree topology of NJ data was completed by performing 1,000 re-samplings and marking the branching points. The Poisson correction method [27] was applied to calculate the evolutionary distances. The two-dimensional structure of Ahg943 and *Zg*AhgB was analyzed using the NetSurfP-2.0 [28] program server (http://www.cbs.dtu.dk/services/NetSurfP/).

## Results

### *In silico* Analysis of Ahg943

**Fig. 2:**
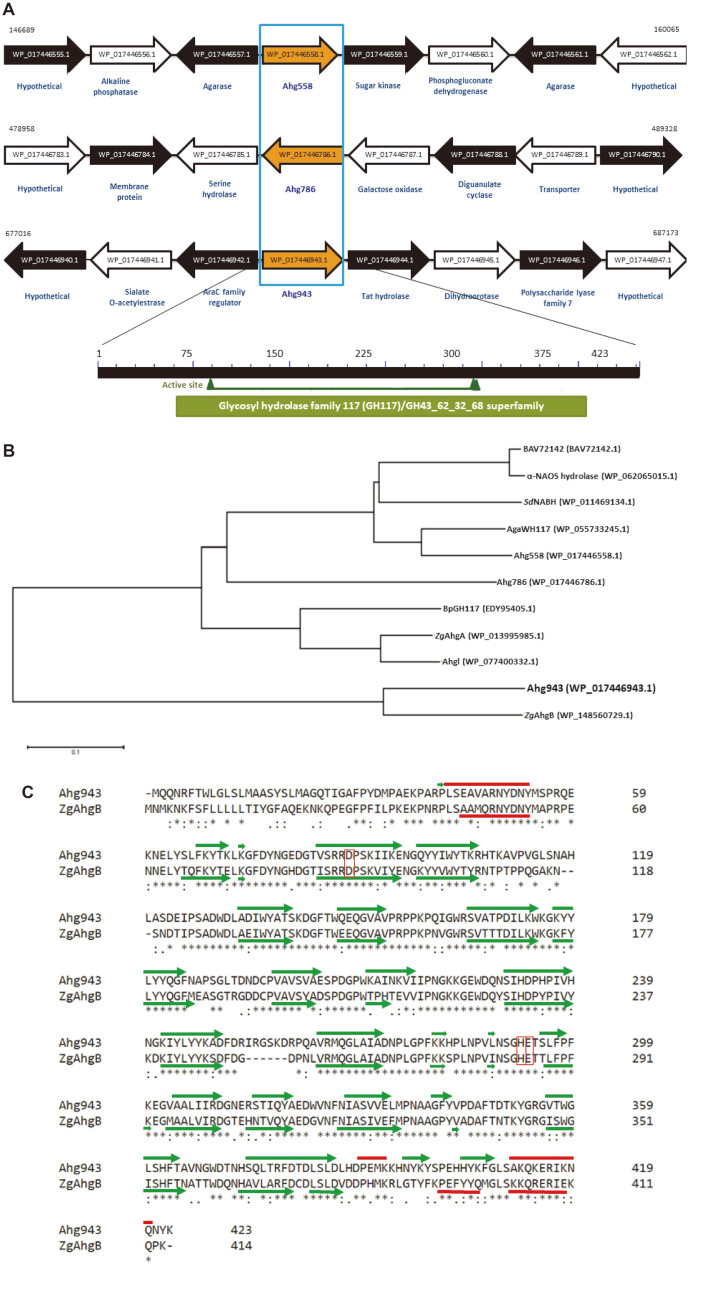
Gene arrangement, phylogenetic analysis, and secondary structure of Ahg943 from *G. joobiniege* G7. (**A**) Gene arrangement of Ahg943 compared to Ahg558 and Ahg786 in the *G. joobiniege* G7 genome and the distribution of the conserved domain. The nucleotide numbers of the chromosomal region used for gene arrangement are presented above both ends of each DNA fragment based on the genomic data of *G. joobiniege* G7. Each ORF is indicated by an arrow with a stop codon at the arrowhead. The NCBI accession number is marked in each arrow with the annotated function in the lower row. Similar to Ahg558 and Ahg786, Ahg943 (WP_017446943.1) has a long conserved domain (cd08992) of the GH117 family spanning between N-61 and D-389 with an e-value of 3.96 xe^-164^. Three putative catalytic residues, D-88, H-292, and E-293, are represented by triangles. (**B**) Phylogenetic tree of α-neoagarooligosaccharide hydrolases, including Ahg943. A phylogenetic tree was constructed by comparing 11 α-neoagarooligosaccharide hydrolases, including Ahg943, identified so far through the neighbor-joining method in MEGA 6. The tree was constructed to have branch lengths in the same units as the evolutionary distances used to infer phylogenetic trees. Sequence ID of each protein is indicated in parentheses. (**C**) Comparison of secondary structure of Ahg943 with ZgAhgB. Two-dimensional structures of Ahg943 and ZgAhgB were constructed using the NetSurfP-2.0 program server (http://www.cbs.dtu.dk/services/NetSurfP/) for comparison. Two proteins were aligned depending on their amino acid sequence using Clustal Omega (https://www.ebi.ac.uk/Tools/msa/clustalo/). The secondary structure of the protein is indicated at the top of Ahg943 and the bottom of ZgAhgB. The alpha-helix structure is shown as a thick line, and the beta-strand is shown as an arrow. The conserved residues constituting the active site are depicted with boxes.

Ahg943 is a polypeptide consisting of 423 amino acids with a calculated molecular mass of 47.96 kDa, and is annotated as a hypothetical protein from *G. joobiniege* G7. Protein BLAST analysis [29] revealed high identity with the biochemically characterized α-NAOSH, *Zg*AhgB (67% identity) from *Zobellia galactanivorans* [30]. However, it showed low identity with the other characterized α-NAOSHs, *Zg*AhgA (40% identity) from *Z. galactanivorans* [31], *Bp*GH117 (35% identity) from the human gut bacterium *Bacteroides plebeius* [32], and *Sd*NABH (36% identity) from *Saccharophagus degradans* 2-40 [33]. It shared low homology with Ahg558 (35%identity) and Ahg786 (36% identity) α-NAOSHs that were identified in *G. joobiniege* G7 [18, 19]. Moreover, comparisons of gene arrangements of three α-NAOSH genes in *G. joobiniege* G7 revealed that there was no open reading frame (ORF) involved in agar metabolism nearest to Ahg943, in contrast to Ahg558 and Ahg786 (Fig. 2A).

A long-conserved domain (cd08992) of the glycosyl hydrolase family 117 (GH117) is located in the region of N-61 and D-389 of Ahg943, with an e-value of 3.96 xe^-164^ (Fig. 2A). Although GH117 shares distant sequence similarity with other families, such as GH43 and GH32, they all belong to the GH43, GH62, GH32, GH68, GH117, and GH130 superfamily and, thus, have a five-bladed beta-propeller domain containing the catalytic acid and base [34, 35]. Therefore, D-88, H-292, and E-293 residues are expected to compose the active sites of Ahg943 [30].

Coinciding with amino acid sequence identity, Ahg943 formed a clade with *Zg*AhgB, which is distinct from other α-NAOSHs, including Ahg558 and Ahg786 (Fig. 2B). The secondary structure of Ahg943 showed a very similar shape, except for the C-terminal region. A helix-turn-helix motif was present in the C-terminus of both proteins, but two beta strands were placed between two helices in the Ahg943 (Fig. 2C).

### Overexpression and Purification of Ahg943

**Fig. 3:**
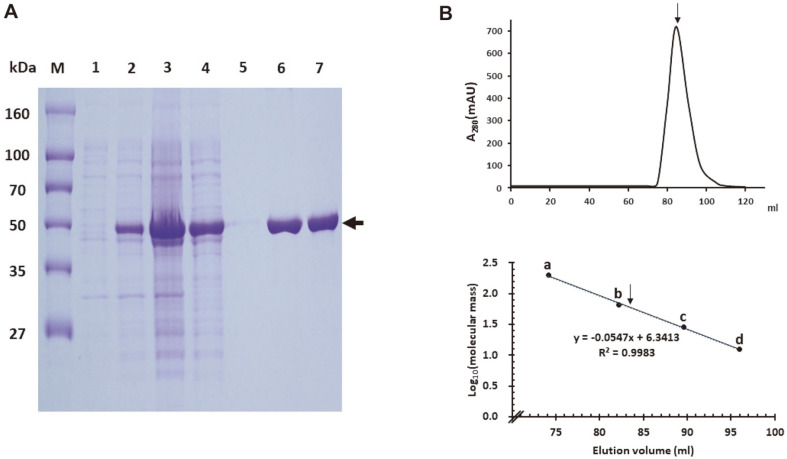
Molecular weight analysis of rAhg943 by sodium dodecyl sulfate-polyacrylamide gel electrophoresis (SDS-PAGE) and gel filtration chromatography. (**A**) SDS-PAGE analysis. rAhg943 with an N-terminus His tag was purified by TALON metal-affinity chromatography. Lanes: M, molecular mass marker; 1, total cell protein before IPTG induction; 2, total cell protein after IPTG induction; 3, pellet protein of IPTG-induced total cell lysate; 4, cell-free extract of IPTG-induced total cell lysate; 5, column washing solution; 6, eluates; 7, eluate after dialysis. The Ahg943 protein is indicated by an arrow. (**B**) Gel filtration chromatography. (upper) The purified rAhg943 protein was applied onto a Superose 12 10/300 GL column and the elution profile at a flow rate of 0.5 ml/min was monitored at 280 nm (A_280_). (lower) Molecular mass of Ahg943 was calculated from the elution profile of size marker proteins: position a, β-amylase (200 kDa); position b, bovine serum albumin (66 kDa); position c, bovine carbonic anhydrase (29 kDa); position d, horse cytochrome c (12.4 kDa). The position of the elution peak for rAhg943 is indicated by an arrow.

The recombinant protein, rAhg943, was purified from a cell-free extract of *E. coli* ER2566/pET28(a)-Ahg943 by TALON affinity chromatography. SignalP 4.1 (http://www.cbs.dtu.dk/services/SignalP-4.1/) predicted that Ahg943 has a signal peptide (1-22) at the N-terminal and, thus, the recombinant protein was designed to have 21 additional amino acids, including 6xHis-tag originating from a vector instead of a signal peptide. The expressed rAhg943 protein showed a single band on SDS-PAGE analysis, consistent with a calculated molecular weight of 47.89 kDa (Fig. 3A).

Gel filtration chromatography using a Superose 12 10/300 GL column revealed that that Ahg943 had an apparent molecular weight of 59.36 kDa, indicating it as a monomer (Fig. 3B). Therefore, Ahg943 is the first α-NAOSH to exist as a monomer in that all α-NABH/α-NAOSH, whose biochemical properties have been identified so far, are dimers or multimers.

### Enzymatic Properties of rAhg943

**Fig. 4:**
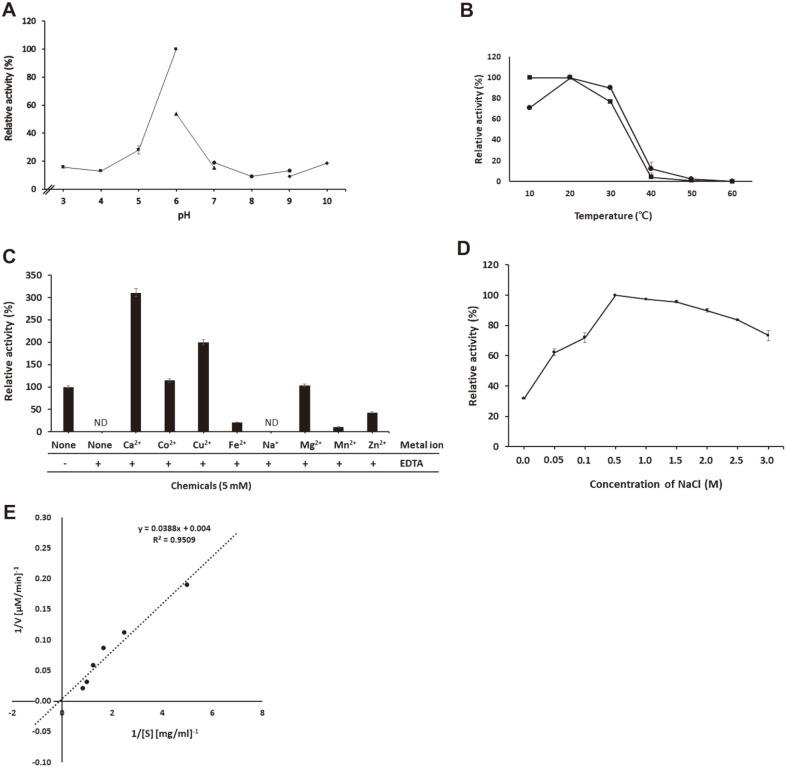
Enzymatic characteristics of rAhg943 toward neoagarobiose. (**A**) Effect of pH. The enzyme reaction was performed in 50 mM citrate buffer (pH 3.0-6.0), MOPS buffer (pH 6.0-7.0), Tris-HCl buffer (pH 7.0-9.0), and glycine buffer (pH 9.0-10.0) at 20°C for 5 h, and measured by the DNS method. (**B**) Effect of temperature. The temperature profile of rAhg786 activity was measured in 50 mM citrate buffer (pH 6.0), containing 5 mM NaCl in the range of 10°C to 60°C for 5 h. The temperature stability of the enzyme was determined after pre-incubation at temperatures ranging from 10°C to 60°C for 1 h. -●-, optimum temperature; -■-, thermostability. In (**A**) and (**B**), the highest enzyme activity was set to 100%, and for all others, the relative activity was calculated. (**C**) Effect of metal ions and EDTA. The effect of metal ions and EDTA on enzyme activity was determined in 50 mM citrate buffer (pH 6.0) in the presence of each chemical (final concentration of 5 mM) at 20°C for 5 h. The relative activities were calculated by considering the enzyme activity without chemicals as 100%. ND, not detected. (**D**) Effect of NaCl concentration. The effect of NaCl on enzyme activity was measured under the same conditions in 50 mM citrate buffer (pH 6.0) in a range of 0-3 M NaCl. The relative activities were calculated by considering the highest enzyme activity of 100%. (**E**) Determination of the kinetic parameters. Lineweaver-Burk plots were used to determine the kinetic parameters of Ahg943 acting on neoagarobiose. In (**A**) and (**E**), all data shown are mean values from at least three replicates.

In the preliminary experiment, the purified rAhg943 showed enzyme activity hydrolyzing neoagarobiose, which led us to investigate its enzymatic properties toward neoagarobiose. In the pH profile experiment, rAhg943 showed maximum activity at pH 6 and drastically decreased activity at pH 5 (28% of the maximum activity) and pH 7 (18% of the maximum activity), indicating a narrow optimum pH range (Fig. 4A). Therefore, all the following experiments were performed in 50 mM citrate buffer at pH 6.

The rAhg943 showed maximum activity at 20°C and maintained its activity at 30°C (90% of the maximum activity), but lost most of its activity above 40°C. Interestingly, it maintained enzyme activity at 10°C (71% of the maximum). Although it was quite stable between 10°C and 20°C, it lost 23% of enzyme activity after heat treatment at 30°C for 1 h (Fig. 4B).

The addition of 0.5 mM EDTA completely abolished the enzyme activity of rAhg943, indicating that it needed a cofactor (Fig. 4C). Monovalent ions, such as Na^+^ and K^+^, did not affect the enzyme activity, but divalent cations, such as Ca^2+^, Co^2+^, Cu^2+^, Mg^2+^, Fe^2+^, Mn^2+^, and Zn^2+^, remarkably enhanced the enzyme activity of rAhg943 at a concentration of 5 mM (data not shown). Therefore, the ability of metal ions to restore the inhibitory effects of EDTA treatment was tested. As a result, Ca^2+^, Co^2+^, Cu^2+^, and Mg^2+^ ions were found to restore the inhibitory effects, and Ca^2+^ remarkably enhanced the enzyme activity 3.1 times higher than the positive control without EDTA (Fig. 4C). This strongly indicates that Ca^2+^ is a cofactor for rAhg943.

Ahg943 was expected to be a secretory monomeric enzyme in seawater, which led us to test its stability depending on salt concentration. The enzyme activity increased depending on NaCl concentration and reached a maximum level at 0.5 M NaCl. In addition, it could maintain enzyme activity above 73% of the maximum activity at 3M NaCl, indicating that it is a high-salt-adapted enzyme (Fig. 4D).

The kinetic parameters, *K*_m_ and *V*_max_, of rAhg943 toward neoagarobiose were 9.7 mg/ml and 250 μM/min (3 U/mg), respectively (Fig. 4E). Of the three α-NAOSHs reported from *G. joobiniege* G7, the *K*_m_ and *V*_max_ values of Ahg786 [19] are 4.5 mg/mL and 1.33 U/mg, respectively, and those of Ahg558 [18] are 8.01 mg/mL and 133.33 U/mg, respectively. Therefore, the *K*_m_ and *V*_max_ values of Ahg943 are intermediate between Ahg786 and Ahg558, and the reaction rate of Ahg558 is considered to be the most excellent.

### Mode of Action

**Fig. 5:**
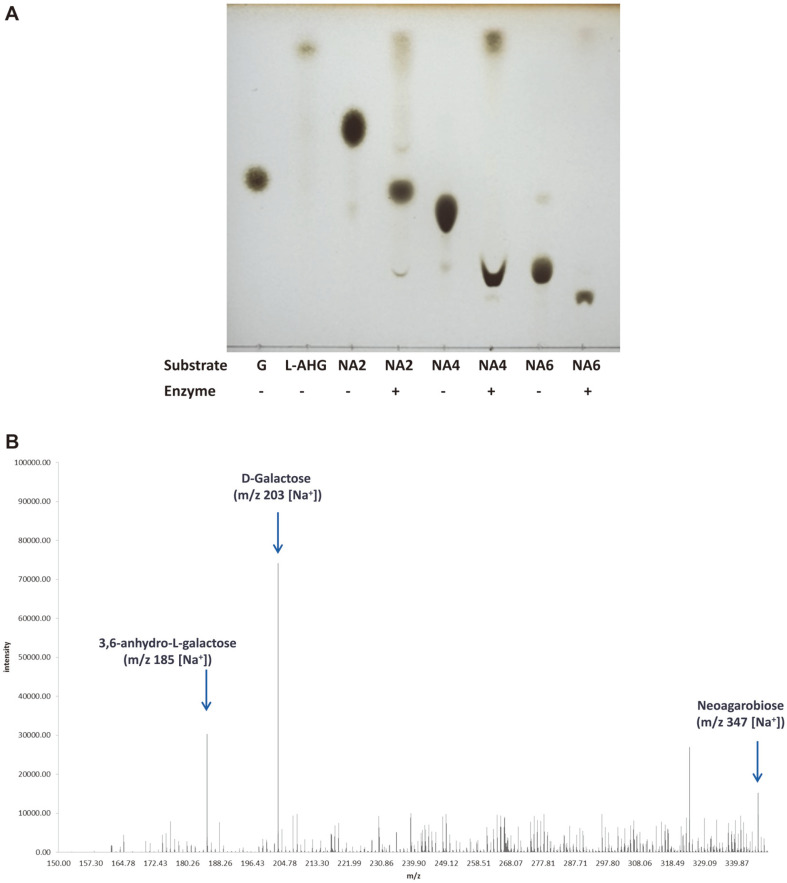
Analysis of degradation products of neoagarooligosaccharides by rAhg943. (**A**) Thin-layer chromatography (TLC). The hydrolyzates of neoagarooligosaccharides by rAhg943 were analyzed by TLC. The hydrolyzates (20°C, 24 h) were loaded onto TLC silica gel plates (60G F_254_) and developed with a solvent (n-butanol:ethanol:water = 3:1:1, v/v). The separated spots were visualized by spraying with 20% (v/v) sulfuric acid in ethanol then heating at 120°C for 15 min. G, Dgalactose; L-AHG, 3,6-anhydro-L-galactose, NA2, neoagarobiose; NA4, neoagarotetraose; NA6, neoagarohexaose. (**B**) Mass spectrometry of neoagarobiose hydrolyzate by rAhg943. The hydrolyzate of neoagarobiose by rAhg943 was prepared as described in (a). The methanol extract of the dried sample was analyzed using LC-TOF-MS (JMS-T100LP 4G, JEOL Ltd, Japan). The peaks for molecular ions at m/z 203 (M+Na)^+^, m/z 185 (M+Na)^+^, and m/z 347 (M+Na)^+^ corresponding to Dgalactose, 3,6-anhydro-l-galactose, and neoagarobiose, respectively, are indicated by arrows. Mass spectrometry for standard neoagarooligosaccharides, neoagarotetraose and neoagarohexaose hydrolyzates by rAhg943 are presented in Figs. S1, S2, and S3.

TLC analysis of the neoagarobiose hydrolyzates by rAhg943 showed two spots corresponding to L-AHG and G (Fig. 5A). The hydrolyzates of neoagarotetraose showed two spots, probably corresponding to L-AHG and neoagarotriose. Moreover, the hydrolyzates of neoagarohexaose also showed two spots, probably corresponding to L-AHG and neoagaropentaose.

The reaction products were further analyzed by mass spectrometry. As expected, the major products of neoagarobiose hydrolyzate had molecular ions at m/z of 185 (M+Na)^+^ and 203 (M+Na)^+^, corresponding to L-AHG and G, respectively (Fig. 5B). The neoagarotetraose hydrolyzate yielded two peaks with molecular ions at *m/z* of 185 (M+Na)^+^ and 509 (M+Na)^+^, corresponding to L-AHG and neoagarotriose, respectively (Figs. S1 and S2). The neoagarohexaose hydrolyzate gave molecular ions at m/z of 185 (M+Na)^+^ and 815 (M+Na)^+^, corresponding to L-AHG and agaropentaose, respectively, (Figs. S1 and S3). The results of TLC and mass analyses indicated that rAhg943 is an α-NAOSH that acts on NAOSs (neoagarobiose, neoagarotetraose, and neoagarohexaose) and releases a common sugar molecule, L-AHG, by hydrolyzing the α-1,3 glycosidic bond from the non-reducing end.

## Discussion

In this study, we identified a unique α-NAOSH Ahg943 classified into GH family 117. GH117 members have distinct N- and C-terminal regions from the members of GH32 and GH43 and have mainly α-NAOSH (E.C. 3.2.1.-) or α-NABH (E.C. 3.2.1.-) activity. To date, only 12 proteins have been functionally characterized among the 368 GH117 members. Among them, *Bp*GH117 from *B. plebeius* [32], *Sd*NABH from *S. degradans* 2-40 [33], and *Zg*AhgA [31] and *Zg*AhgB [30] from *Z. galactanivorans* were studied in their 3D structures. Each monomer represents a funnel-like topology constructed by the five-bladed β-propeller folds, indicating the exo-mode of action of enzyme activity. Three catalytic acidic residues, acting as a base (nucleophile) and acid (proton donor), are positioned at the bottom of this pocket [35]. These common features are also well conserved in Ahg943.

All α-NAOSHs reported so far form dimers or multimeric complexes located in extracellular or intracellular fractions at similar frequencies judging from the presence of signal peptides. Ahg943 is expected to be an extracellular protein in that it has a signal peptide but is distinguished from all α-NAOSHs as it exists as a monomer.

Based on three-dimensional structural analyses, the four α-NAOSHs mentioned above have a common helix-turn-helix domain at their N-terminal region (residues 33-65 in *Bp*GH117, residues 9-29 *Sd*NABH, 38-71 in *Zg*AhgA, and 28-61 in *Zg*AhgB), which contributes to dimerization by swapping the N-terminal extension between two monomers through electrostatic and hydrophobic interactions. The C-terminal region also seems to contribute to stabilize the dimeric structure. For example, the C-termini of *Bp*GH117s (residues 386-401) and SdGH117 (residues 350-371) formed a long loop structure between β-strands that interacted with the surface of the partner to form the dimer, which increased the contact area between the monomers as the N-terminal helix-turn helix [32, 33]. Instead, a helix-turn-helix domain at the C-terminus (residues 386-402) was expected to play a similar role in the dimerization of *Zg*AhgB [30]. However, no such special secondary structure was found at the C-terminus of *Zg*AhgA [31]. When the secondary structure of Ahg943 was compared to that of the phylogenetically closest *Zg*AhgB, one main difference was found in the C-terminal regions; a helix-turn-helix motif was present in the C-terminus of both proteins, but two beta strands were placed between two helices in Ahg943. This structural difference may result in the monomeric property of Ahg943, which needs further validation.

Although the optimum pH of Ahg943 was comparable to that of other α-NAOSHs, the temperature profile demonstrated its unique cold-adapted property. To date, we have identified four β-agarases [36-39] and two α-NAOSHs, Ahg786 [18] and Ahg558 [19], from *G. joobiniege* G7, and all were characterized by unique cold-adapted properties.

Ahg943 activity was maximum at 0.5 M NaCl and maintained above 73% of the maximum at 3M NaCl, indicating that it is a high salt-adapted enzyme. This unique feature of high salt tolerance may also endow great advantages in industrial applications. To date, one GH16 β-agarase (CaAga1) has been reported to have high salt tolerance, maintaining more than 70% of the maximum activity at 2 M NaCl from the marine bacterium *Cellulophaga algicola* DSM 14237 [40]. Therefore, if a variety of halophilic enzymes are identified and used in proper combinations, it will be a much more efficient process for processing marine algal biomass containing high concentrations of salts.

The effects of metal ions on α-NAOSHs seem to be variable. Previously, we reported that Ahg558 and Ahg786 are Mn^2+^-dependent enzymes [18, 19]. Crystallographic analysis of α-NAOSHs, except *Sd*NABH [33], was modeled into a conserved metal-binding pocket located at the bottom of the active site pocket with divalent metal ions, such as Zn^2+^ for *Zg*AhgA [31], Ca^2+^ for *Zg*AhgB [30], or Mg^2+^ ions for *Bp*GH117 [32], but the dependence of the enzyme activity on each metal ion is not yet fully understood. In this study, we clearly proved that that Ahg943 is a Ca^2+^-dependent hydrolase. Therefore, when interpreted together with the results of 3D structural analysis of the phylogenetically closest *Zg*AhgB [30], Ca^2+^ is expected to bind to the metal-binding pocket of Ahg943 and promote enzymatic activity. Moreover, it is expected that D-88, H-292, and E-293 constitute the active site of Ahg943, where H-292 and D-88 are catalytic and general bases, respectively, and E-293 is expected to modulate p*Ka* near the general base [30].

GH117 α-NAOSH catalyzes the final stage of agar decomposition and is an essential step for decomposing and converting agar into other chemicals or bioenergy. However, there are few biochemically identified enzymes so far, and there have been no studies regarding a thermophilic α-NAOSH that hydrolyzes agar in the sol state. The psychrophilic α-NAOSH described in this paper is also thought to be useful for the development of low-temperature clean processes for agar decomposition. Therefore, to meet the current and future industrial applications of marine biomass for functional food and cosmetic, biochemical, and bioenergy industries, more diverse types of α-NAOSH need to be discovered in future.

## Supplemental Materials

Supplementary data for this paper are available on-line only at http://jmb.or.kr.
