# Supplementary material for: Molecular Characterization of a Novel 1,3-α-3,6-Anhydro-L-Galactosidase, Ahg943, with Cold- and High-Salt-Tolerance from Gayadomonas joobiniege G7
Source: J Microbiol Biotechnol. 2020 Aug 28;30(11):1659–69. doi: 10.4014/jmb.2008.08017 (PMC9728383; doi:10.4014/jmb.2008.08017)

J. Microbiol. Biotechnol.
https://doi.org/10.4014/jmb.2008.08017

Supplementary Figure 1. Mass spectrometry of neoagarooligosaccharides mixture

Standard neoagarooligosaccharides substrates containing neoagarobiose, neoagarotetraose, and neoagarohexaose was dissolved in methanol and analyzed using a LC-TOF-MS (JMS-T100LP 4G, JEOL Ltd, Japan). The peaks for molecular ions at m/z 347 $(M+Na)^+$, m/z 648 $(M+Na)^+$, and m/z 959 $(M+Na)^+$ corresponding to neoagarobiose, neoagarotetraose, and neoagarohexaose, respectively, are indicated by arrows.

Supplementary Figure 1

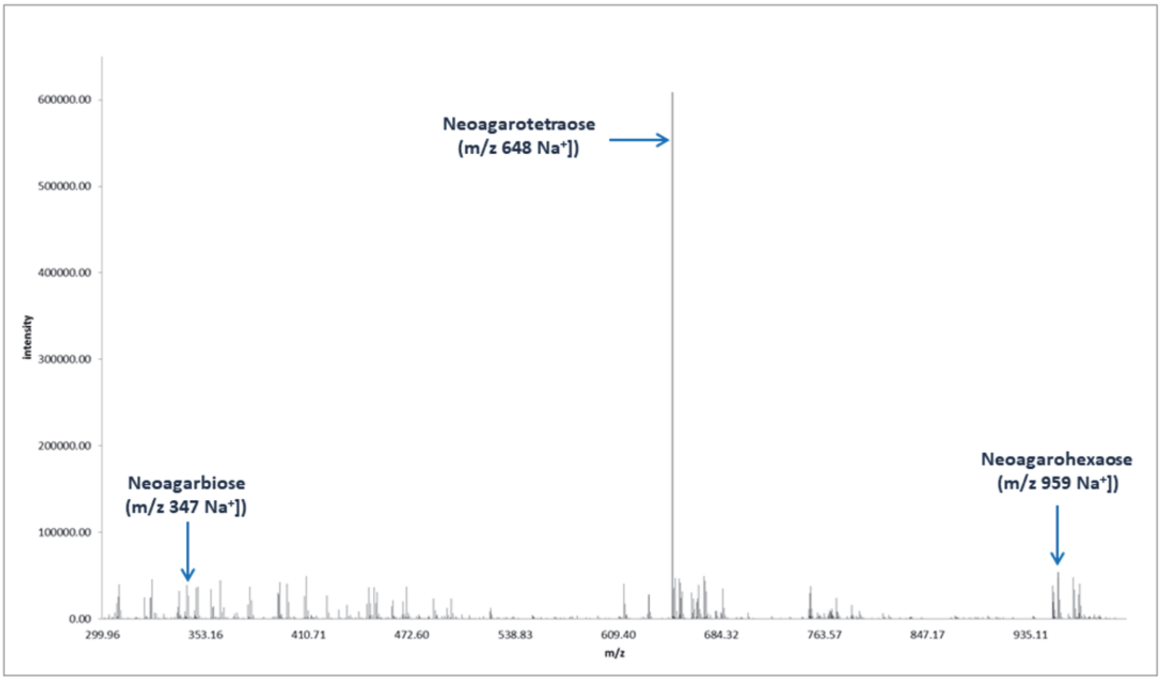

Supplementary Figure 2. Mass spectrometry of neoagarotetraose hydrolysate by rAhg943

Hydrolyzed product of neoagarotetraose was extracted with methanol and then analyzed using a LC-TOF-MS (JMS-T100LP 4G, JEOL Ltd, Japan). The peaks for molecular ions at m/z 185 $(M+Na)^+$ and m/z 509 $(M+Na)^+$ corresponding to 3,6-anhydro-l-galactose and neoagarotriose, respectively, are indicated by arrows.

Supplementary Figure 2

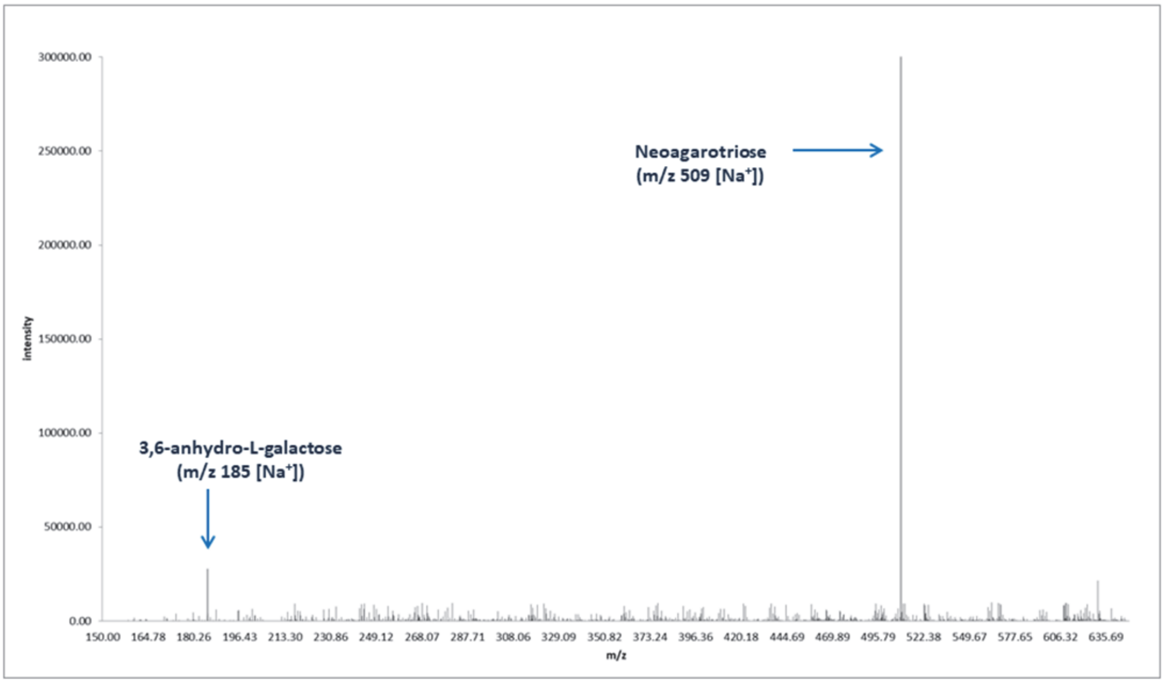

Supplementary Figure 3. Mass spectrometry of neoagarohexaose hydrolysate by rAhg943

Hydrolyzed product of neoagarohexaose was analyzed using an LC-TOF-MS (JMS-T100LP 4G, JEOL Ltd, Japan). The peaks for molecular ions at m/z 185 $(M+Na)^+$ and m/z 815 $(M+Na)^+$ corresponding to 3,6-anhydro-l-galactose and neoagaropentaose, respectively, are indicated by arrows.

Supplementary Figure 3

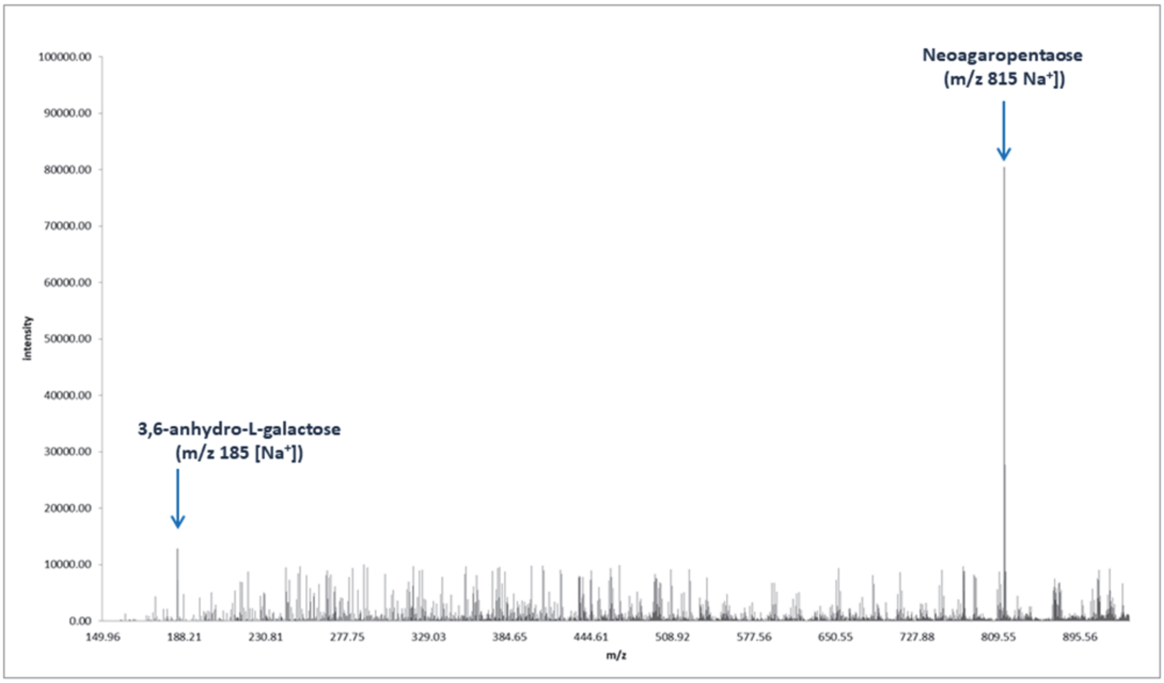

Supplement: Supplementary file 1 [file JMB-30-11-1659-supple.pdf]
